# Four-Objective Optimization of Irreversible Atkinson Cycle Based on NSGA-II

**DOI:** 10.3390/e22101150

**Published:** 2020-10-13

**Authors:** Shuangshuang Shi, Yanlin Ge, Lingen Chen, Huijun Feng

**Affiliations:** 1 Institute of Thermal Science and Power Engineering, Wuhan Institute of Technology, Wuhan 430205, China; shishuangshuang20@163.com (S.S.); geyali9@hotmail.com (Y.G.); huijunfeng@139.com (H.F.); 2 School of Mechanical & Electrical Engineering, Wuhan Institute of Technology, Wuhan 430205, China

**Keywords:** Atkinson cycle, power output, power density, thermal efficiency, ecological function, finite time thermodynamics

## Abstract

Variation trends of dimensionless power density (PD) with a compression ratio and thermal efficiency (TE) are discussed according to the irreversible Atkinson cycle (AC) model established in previous literature. Then, for the fixed cycle temperature ratio, the maximum specific volume ratios, the maximum pressure ratios, and the TEs corresponding to the maximum power output (PO) and the maximum PD are compared. Finally, multi-objective optimization (MOO) of cycle performance with dimensionless PO, TE, dimensionless PD, and dimensionless ecological function (EF) as the optimization objectives and compression ratio as the optimization variable are performed by applying the non-dominated sorting genetic algorithm-II (NSGA-II). The results show that there is an optimal compression ratio which will maximize the dimensionless PD. The relation curve of the dimensionless PD and compression ratio is a parabolic-like one, and the dimensionless PD and TE is a loop-shaped one. The AC engine has smaller size and higher TE under the maximum PD condition than those of under the maximum PO condition. With the increase of TE, the dimensionless PO will decrease, the dimensionless PD will increase, and the dimensionless EF will first increase and then decrease. There is no positive ideal point in Pareto frontier. The optimal solutions by using three decision-making methods are compared. This paper analyzes the performance of the PD of the AC with three losses, and performs MOO of dimensionless PO, TE, dimensionless PD, and dimensionless EF. The new conclusions obtained have theoretical guideline value for the optimal design of actual Atkinson heat engine.

## 1. Introduction

Finite time thermodynamics (FTT) [1,2,3,4,5,6,7] is an effective theoretical tool for performance analysis and optimization of internal combustion engine cycles, and it has made great progress [8,9,10]. It includes optimal performance research [11,12,13,14] and optimal configuration research [15,16,17,18,19,20,21,22,23]. Compared with the Otto cycle, the Atkinson cycle (AC) has higher thermal efficiency (TE) and lower fuel consumption. Some scholars have studied its performance by utilizing FTT. Hou [24] analyzed the relationship between power output (PO) and TE of the endoreversible AC with only heat transfer loss (HTL). Ge et al. [25,26,27,28] analyzed the PO and TE characteristics of the endoreversible [26] AC with HTL, and irreversible [25,27,28] AC with HTL, friction loss (FL), and internal irreversibility loss (IIL) considering the constant specific heat [25], variable specific heat with linear [26,27], and non-linear [28] relations with the temperature of working fluid (WF). Gonca [29] analyzed the effective PO and effective PD characteristics of the irreversible AC with incomplete combustion loss, FL, HTL, and exhaust output loss considering the specific heat varied non-linearly with the WF’s temperature. Ebrahimi et al. [30] analyzed the relationship between the PO and TE of the irreversible AC with HTL, IIL, and FL considering a variable specific heat ratio with non-linear relation with temperature of WF.

When power density (PD; ratio of PO to the maximum specific volume in the cycle) is taken as the optimization goal, a heat engine can increase the TE and reduce the volume of the whole device at the expense of a little decrease of the PO. Without any loss considered, Chen et al. [31] found that a reversible TE of AC under the maximum PD condition was higher than that of under the maximum PO condition, and the size of the engine under the maximum PD condition was smaller. Ust et al. [32] discussed the PD characteristics of irreversible AC with IIL and compared the results with those in Reference [31]. Al-Sarkhi et al. [33] studied the TE of the endoreversible AC when specific heat varied linearly with the WF’s temperature under the maximum PD condition. With the PD as the objective, Gonca [34] optimized the irreversible Dual–Atkinson cycle when specific heat varied non-linearly with the WF’s temperature.

The NSGA-II has been widely used in FTT optimizations. Sadatsakkak et al. [35] optimized the dimensionless PD and TE of endoreversible Braysson cycle by applying the NSGA-II, and compared the optimization results obtained by using different decision-making ways. Dai et al. [36] carried out multi-objective optimization (MOO) on PO, TE, and ecological performance coefficient of a regenerative Stirling engine. Ahmadi et al. [37] carried out MOO on the PD, TE, ecological function (EF) density, and exergy loss density of fuel cell-Braysson combined cycle. Ghasemkhani et al. [38] carried out MOO on the PO, TE, exergy loss, and EF of two-stage endoreversible combined Carnot cycle. Turgut et al. [39] performed MOO on the performance coefficient, exergy efficiency, ecological performance coefficient, thermo-economic optimization, and thermo-ecological optimization functions of irreversible simple air refrigerator. Abedinnezhad et al. [40] carried out MOO on the TE, ecological performance coefficient and EF of irreversible Dual–Miller cycle. Tang et al. [41] and Chen et al. [42] applied the NAGA-II algorithm to perform MOO on the three- and four-objective optimizations for Brayton cycles. Zhang et al. [43] and Sun et al. [44] applied the NAGA-II algorithm to perform MOO on the chemical reactors. Wu et al. [45] performed MOO on the entropy generation rate and total pumping PO of a condenser in the ocean thermal energy conversion (OTEC) system based on the constructal theory and NSGA-II algorithm.

On the basis of the irreversible AC model established in Reference [28], this paper will take the PD as the objective to carry out FTT analysis and performance optimization for the irreversible AC model with constant specific heat of WF, and perform the MOO on the dimensionless PO, TE, dimensionless PD, and dimensionless EF by using the NSGA-II algorithm. The major differences between this paper and References [31,32,33,34] are as followings. Firstly, this paper will consider the HTL, FL, and ILL, whereas Reference [31] did not consider any loss and Reference [32] only considered IIL. Secondly, this paper will consider the constant specific heat with temperature of WF, while References [33,34] considered the variable specific heat with linear [33] and non-linear [34] relations with temperature of WF, respectively. Thirdly, this paper will consider four-objective optimization, but References [31,32,33,34] did not consider multi-objective optimization.

## 2. Irreversible AC Model

**Figure 1:**
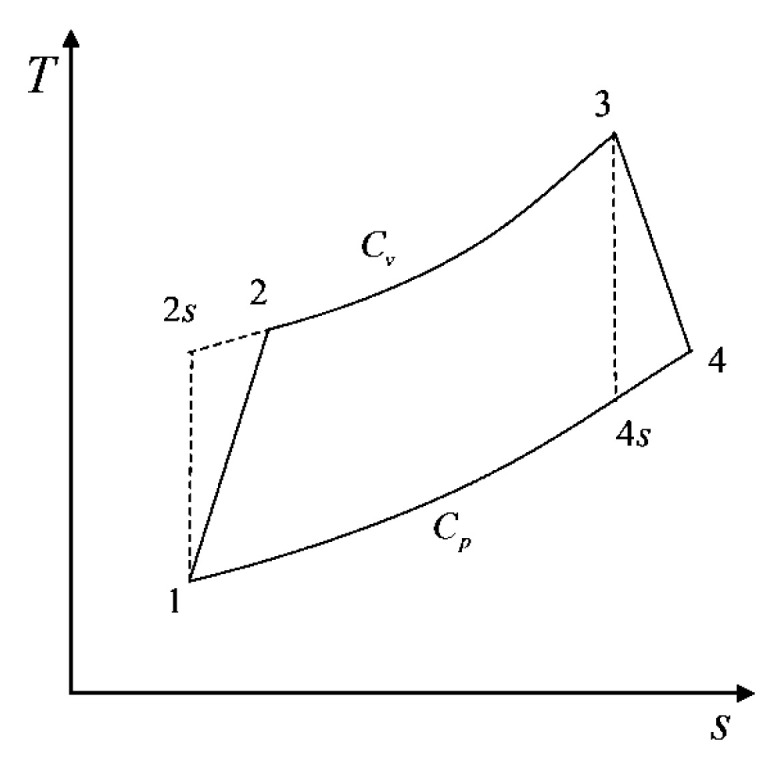
T−S diagram for the Atkinson cycle model.

Figure 1 shows the temperature-entropy (T−S) diagram irreversible AC model [28]. 1→2 and 3→4 are irreversible adiabatic processes, and 1→2s and 3→4s are the corresponding isentropic processes. 2→3 is an endothermic process with constant volume, and 4→1 is an exothermic process with constant pressure.

The WF’s heat absorption rate in the cycle is

(1) $${\dot{Q}}_{in}=\dot{m}C_{v}(T_{3}-T_{2})$$

The WF’s heat release rate in the cycle is

(2) $${\dot{Q}}_{out}=\dot{m}C_{p}(T_{4}-T_{1})$$

where $C_{v}$ ($C_{p}$) is the specific heat of WF at constant volume (pressure) and $\dot{m}$ is the molar flow rate of the WF.

According to References [28,46,47], the compression and expansion efficiencies in two adiabatic processes 1→2 and 3→4 are defined to represent the IIL of the cycle

(3) $$\eta_{c}=\left(T_{2s}-T_{1}\right)/\left(T_{2}-T_{1}\right)$$

(4) $$\eta_{e}=\left(T_{4}-T_{3}\right)/\left(T_{4s}-T_{3}\right)$$

The compression ratio γ and the maximum temperature ratio τ of the AC are defined as:

(5) $$\gamma =V_{1}/V_{2}$$

(6) $$\tau =T_{3}/T_{1}$$

In additional, the entropy change of the working fluid equals zero after a cycle, one has:

(7) $$\Delta S=C_{v}\ln(T_{3}/T_{2s})-C_{p}\ln(T_{4s}/T_{1})=0$$

According to the property of isentropic process, one has:

(8) $$T_{2s}{\left(T_{4s}\right)}^{k}=T_{3}{\left(T_{1}\right)}^{k}$$

From Equations (3)–(8), one has:

(9) $$T_{2}=[(\gamma^{k-1}-1)/\eta_{c}+1]T_{1}$$

(10) $$T_{4}=T_{1}[\tau (1-\eta_{e})+\eta_{e}\tau^{\left(1/k\right)}\gamma^{\left(1/k-1\right)}]$$

For the actual AC, the HTL between cylinder wall and WF cannot be negligible. According to Reference [48], the heat absorption rate in process 2→3 is

(11) $${\dot{Q}}_{in}=A-B[(T_{2}+T_{3})/2-T_{0}]$$

where A and B are the heat released rate by fuel combustion and the HTL coefficient, respectively, and $T_{0}$ is ambient temperature.

Equation (11) shows that the cycle heat absorption rate includes two parts. The total heat absorption rate by WF is equal to the difference between the heat release rate by fuel combustion and the HTL rate. Therefore, the HTL rate is:

(12) $${\dot{Q}}_{leak}=B_{1}(T_{2}+T_{3}-2T_{0})$$

where $T_{0}$ is the ambient temperature and $B_{1}=B/2$.

In the actual AC, the FL between the piston and cylinder wall should also be considered. According to the treatment method of Otto cycle in Reference [49], the friction force is

(13) $$f_{\mu }=-\mu v=-\mu dx/dt$$

where μ and x are the friction coefficient and the displacement of piston, respectively.

The FL power is obtained

(14) $$P_{\mu }=dW_{\mu }/dt=-\mu {\left(dx/dt\right)}^{2}=-\mu v^{2}$$

The piston average speed $\bar{v}$ is used to replace the piston movement speed v,

(15) $$\bar{v}=(x_{1}-x_{2})/\Delta t_{12}=x_{2}(\gamma -1)/\Delta t_{12}$$

where $x_{1}$ is the position of the piston at the maximum volume, $x_{2}$ is the position of the piston at the minimum volume, and $\Delta t_{12}$ is the time consumed by the power stroke.

The cycle PO is obtained:

(16) $$P={\dot{Q}}_{in}-{\dot{Q}}_{out}-P_{\mu }=\dot{m}C_{v}[(T_{3}-T_{2})-k(T_{4}-T_{1})]-b{\left(\gamma -1\right)}^{2}$$

where $b=\mu x_{2}^{2}/{\left(\Delta t_{12}\right)}^{2}$.

The TE is written as:

(17) $$\eta =\frac{P}{{\dot{Q}}_{in}+{\dot{Q}}_{leak}}=\frac{\dot{m}C_{v}[k(T_{1}-T_{4})+(T_{3}-T_{2})]-b{\left(\gamma -1\right)}^{2}}{\dot{m}C_{v}(T_{3}-T_{2})+B_{1}(T_{2}+T_{3}-2T_{0})}$$

According to Reference [31], the PD is defined as:

(18) $$P_{d}=P/v_{4}$$

According to the $v_{1}/v_{4}=T_{1}/T_{4}$, one has:

(19) $$P_{d}=P/[v_{1}(T_{4}/T_{1})]$$

There are HTL, FL, and IIL in the actual irreversible AC. The entropy generation rate caused by HTL and FL are, respectively:

(20) $$\sigma_{q}=B_{1}(T_{2}+T_{3}-2T_{0})[1/T_{0}-2/(T_{2}+T_{3})]$$

(21) $$\sigma_{\mu }=P_{\mu }/T_{0}=b{\left(\gamma -1\right)}^{2}/T_{0}$$

The entropy generation rate due to the IIL is calculated by the entropy increase rates in processes 2s→2 and 4s→4

(22) $$\sigma_{2s\to 2}=\dot{m}C_{v}\ln(T_{2}/T_{2s})$$

(23) $$\sigma_{4s\to 4}=\dot{m}C_{p}\ln(T_{4}/T_{4s})$$

After the power stroke, the WF is discharged to the environment in the exhaust stroke. The entropy generation rate caused in this process is:

(24) $$\sigma_{pq}=\dot{m}\int_{T_{1}}^{T_{4}}C_{p}dT(1/T_{0}-1/T)=\dot{m}k[C_{v}(T_{4}-T_{1})/T_{0}-C_{v}\ln(T_{4}/T_{1})]$$

The total entropy generation rate of the AC is:

(25) $$\sigma =\sigma_{q}+\sigma_{\mu }+\sigma_{2s\to 2}+\sigma_{4s\to 4}+\sigma_{pq}$$

According to the Reference [50], the EF is defined as:

(26) $$E=P-T_{0}\sigma$$

According to the treatment method of reversible Atkinson cycle by Chen et al. [31], the dimensionless PO, dimensionless PD, and dimensionless EF are defined as, respectively:

(27) $$\bar{P}=P/P_{\max}$$

(28) $${\bar{P}}_{d}=P_{d}/{\left(P_{d}\right)}_{\max}$$

(29) $$\bar{E}=E/E_{\max}$$

when γ, $T_{1}$, and τ are given, the temperatures at each state point can be solved, and then the numerical solutions of $\bar{P}$, η, ${\bar{P}}_{d}$, and $\bar{E}$ can be obtained.

## 3. Power Density Analysis and Optimization

Based on References [26,27,32], the following parameters are determined: B=2.2 W/K, $T_{0}=300\;K$, $T_{1}=350\;K$, $\dot{m}=1\;\mathrm{mol}/s$, $C_{v}=20.78\;J/(\mathrm{mol}\cdot K)$, k=1.4, τ=4.28−6.28, and b=20 W.

**Figure 2:**
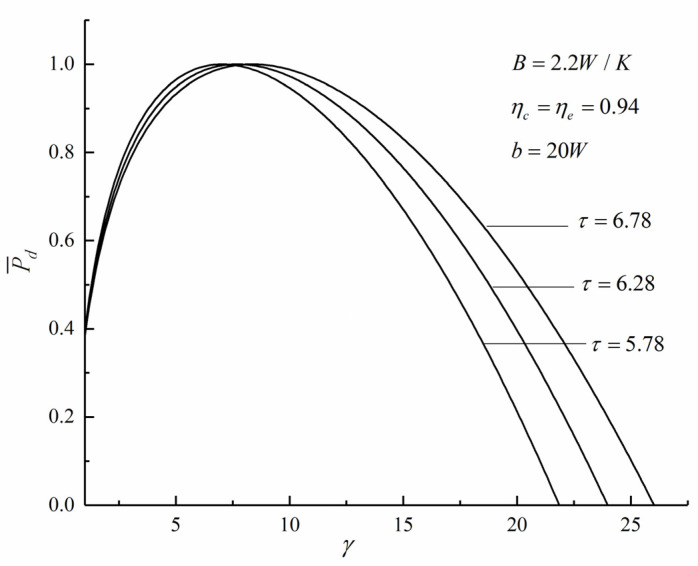
Effect of τ on ${\bar{P}}_{d}$ versus γ.

**Figure 3:**
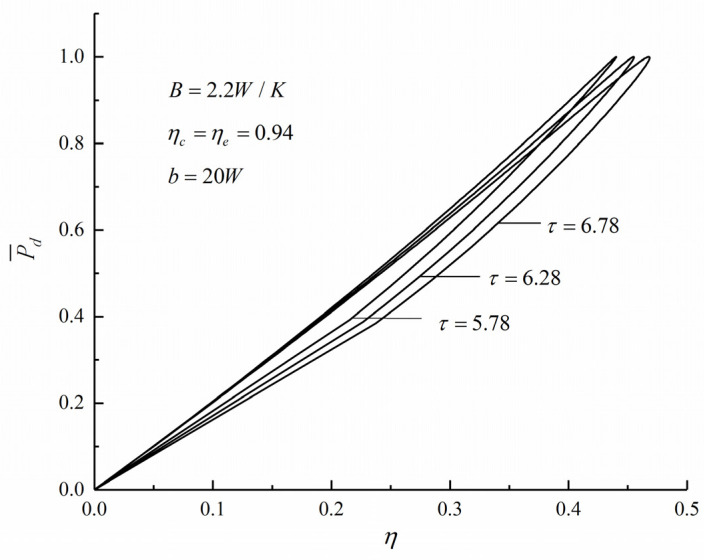
Effect of τ on ${\bar{P}}_{d}$ versus η.

Figure 2 and Figure 3 show the influence of the temperature ratio (τ) on dimensionless PD and compression ratio (${\bar{P}}_{d}-\gamma$) and dimensionless PD and TE (${\bar{P}}_{d}-\eta$) characteristics, respectively. The shape of the ${\bar{P}}_{d}-\gamma$ curve is parabolic-like one, and there is an optimal value $\gamma_{{\bar{P}}_{d}}$ which makes the ${\bar{P}}_{d}$ reach the maximum value ${\left({\bar{P}}_{d}\right)}_{\max}$. The shape of the ${\bar{P}}_{d}-\eta$ curve is a loop-shape one back to the origin, and there is another optimal value $\gamma_{\eta }$ which makes the η reach the maximum value $\eta_{\max}$. With the increase of τ, the $\gamma_{{\bar{P}}_{d}}$ and the $\eta_{{\bar{P}}_{d}}$ increase. The numerical calculations show that when τ increases from 5.78 to 6.78, the $\eta_{{\bar{P}}_{d}}$ increases from 0.4402 to 0.4684 and increase by 6.41%.

**Figure 4:**
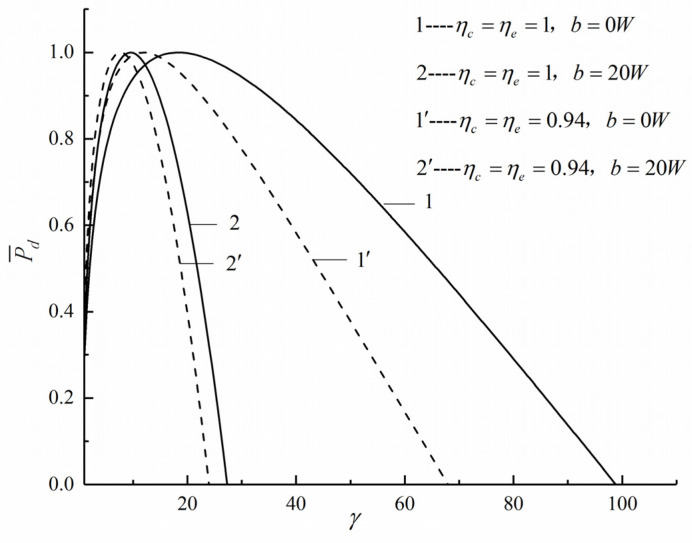
Effects of $\eta_{c}$, $\eta_{e}$ and b on ${\bar{P}}_{d}$ versus γ.

Figure 4 shows the characteristics relationship of ${\bar{P}}_{d}$ and γ with different $\eta_{c}$, $\eta_{e}$, and b. Curves 1 and $1^{\prime }$ show the effect of the IIL on ${\bar{P}}_{d}$ without FL. Curves 2 and $2^{\prime }$ show the effect of the IIL on ${\bar{P}}_{d}$ with FL. The $\gamma_{{\bar{p}}_{d}}$ decreases with the increase of the IIL whether the FL is considered or not. Curves 1 and 2 show the effect of the FL on ${\bar{P}}_{d}$ without IIL. Curves $1^{\prime }$ and $2^{\prime }$ show the effect of the FL on ${\bar{P}}_{d}$ with IIL. The $\gamma_{{\bar{p}}_{d}}$ decreases with the increase of the FL whether the IIL is considered or not.

**Figure 5:**
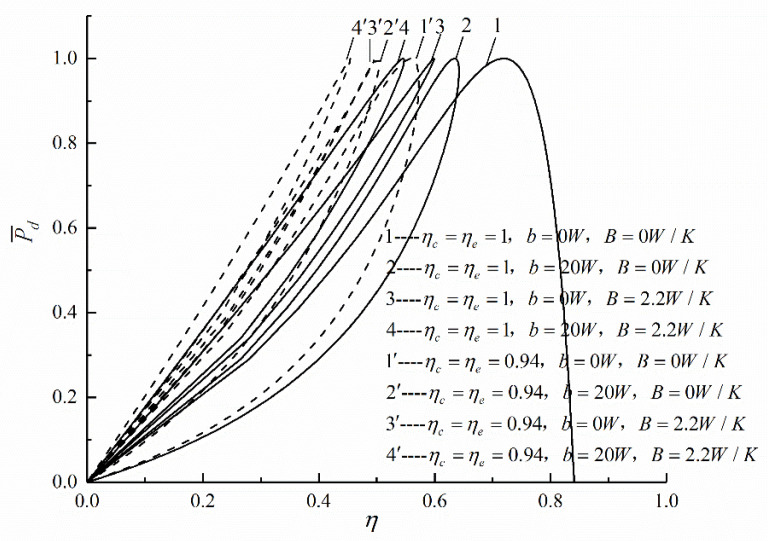
Effects of $\eta_{c}$, $\eta_{e}$, B and b on ${\bar{P}}_{d}$ versus η.

Figure 5 shows the influences of $\eta_{c}$, $\eta_{e}$, B, and b on ${\bar{P}}_{d}-\eta$ characteristics. The curve 1 reflects the ${\bar{P}}_{d}-\eta$ characteristic when the cycle is completely reversible, and the shape of curve is a parabolic-like one ($\eta_{{\bar{P}}_{d}}\neq 0$ but ${\left({\bar{P}}_{d}\right)}_{\eta }=0$). The other curves reflect the ${\bar{P}}_{d}-\eta$ characteristics when one or more irreversibility is considered, the shape of curve is a loop-shaped one back to the origin (both the $\eta_{{\bar{P}}_{d}}$ and ${\left({\bar{P}}_{d}\right)}_{\eta }$ are not zero). Comparing curves 1 and $1^{\prime }$, 2 and $2^{\prime }$, 3 and $3^{\prime }$, as well as 4 and $4^{\prime }$ in Figure 5, one can see that $\eta_{{\bar{P}}_{d}}$ increases with the decrease of IIL ($\eta_{c}$ and $\eta_{e}$ are increased). The numerical calculations show that when B=2.2 W/K and b=20 W, and $\eta_{c}$ and $\eta_{e}$ increases from 0.94 to 1, $\eta_{{\bar{P}}_{d}}$ increases from 0.4551 to 0.5456, and increases by 19.89%. Comparing curves 1 and 2, 3 and 4, $1^{\prime }$ and $2^{\prime }$, as well as $3^{\prime }$ and $4^{\prime }$ in Figure 5, one can see that $\eta_{{\bar{P}}_{d}}$ decreases with the increase of FL. The numerical calculations show that when $\eta_{c}=\eta_{e}=0.94$ and B=2.2 W/K, and b increases from 0 W to 20 W, and $\eta_{{\bar{P}}_{d}}$ decreases from 0.5021 to 0.4551, and decreases by 9.36%. Comparing curves 1 and 3, 2 and 4, $1^{\prime }$ and $3^{\prime }$, as well as $2^{\prime }$ and $4^{\prime }$ in Figure 5, one can be seen that $\eta_{{\bar{P}}_{d}}$ decreases with the increase of HTL. The numerical calculations show that when $\eta_{c}=\eta_{e}=0.94$ and b=20 W, and B increases from 0 W/K to 2.2 W/K, $\eta_{{\bar{P}}_{d}}$ decreases from 0.4970 to 0.4551, and decreases by 8.43%.

**Figure 6:**
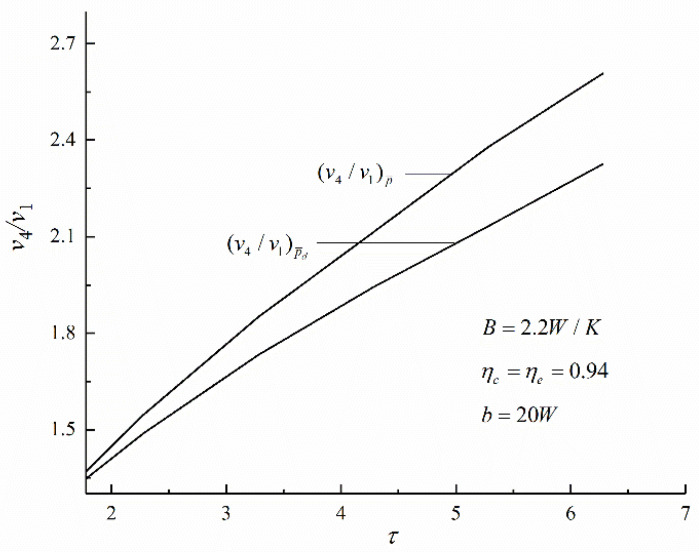
Variations of the maximum specific volume ratio $v_{4}/v_{1}$ with the maximum cycle temperature ratio τ.

Under the conditions of maximum PO ($P_{\max}$) and maximum PD (${\left(P_{d}\right)}_{\max}$), Figure 6 shows the relations of maximum specific volume ratio $v_{4}/v_{1}$ and τ. The ${\left(v_{4}/v_{1}\right)}_{{\bar{P}}_{d}}$ is smaller than the ${\left(v_{4}/v_{1}\right)}_{\bar{P}}$ when τ is a constant. The numerical calculations show that when τ=6.28, ${\left(v_{4}/v_{1}\right)}_{\bar{P}}$ is 2.61 and ${\left(v_{4}/v_{1}\right)}_{{\bar{P}}_{d}}$ is 2.32. Compared with ${\left(v_{4}/v_{1}\right)}_{\bar{P}}$, ${\left(v_{4}/v_{1}\right)}_{{\bar{P}}_{d}}$ decreases by 11.1%. The size of the AC engine is smaller under the condition of ${\left(P_{d}\right)}_{\max}$.

**Figure 7:**
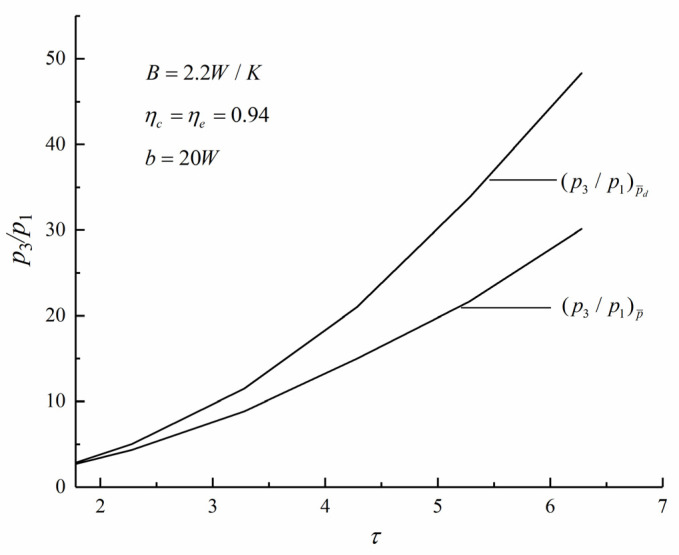
Variations of the maximum pressure ratio $p_{3}/p_{1}$ with the maximum cycle temperature ratio τ.

Under conditions of $P_{\max}$ and ${\left(P_{d}\right)}_{\max}$, Figure 7 shows the relations of maximum pressure ratio $p_{3}/p_{1}$ and τ. It can be seen that ${\left(p_{3}/p_{1}\right)}_{\bar{P}}$ is always smaller than ${\left(p_{3}/p_{1}\right)}_{{\bar{P}}_{d}}$ when τ is a constant. It means that the decrease of the AC engine size is accompanied by the increase of maximum pressure ratio in the cycle.

**Figure 8:**
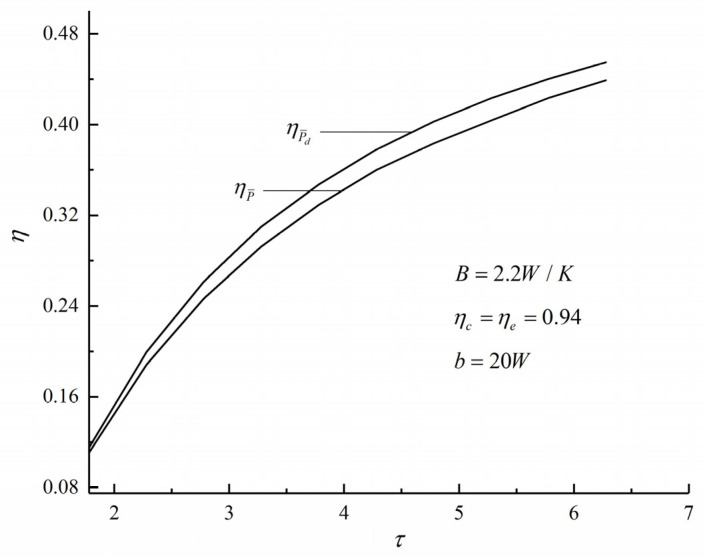
Variations of various thermal efficiency η with the maximum cycle temperature ratio τ.

Figure 8 shows the relations of the η and τ. One can see that when there are three losses, $\eta_{P_{d}}$ is larger than $\eta_{P}$. When τ=6.28, $\eta_{P}$ is 0.4389 and $\eta_{P_{d}}$ is 0.4549. Compared with $\eta_{P}$, $\eta_{P_{d}}$ increases by 3.65%.

Figure 6 and Figure 8 show that when there are three losses, compared with the maximum PO condition, $\eta_{P_{d}}$ at the maximum PD condition increases by 3.65%, while ${\left(v_{4}/v_{1}\right)}_{{\bar{P}}_{d}}$ at the maximum PD condition decreases by 11.1%. The results show that the TE is larger and the size of the heat engine is smaller when the ${\left(P_{d}\right)}_{\max}$ is taken as the objective.

## 4. Four Objective Optimization and Decision-Making Based on NSGA-II Algorithm

**Figure 9:**
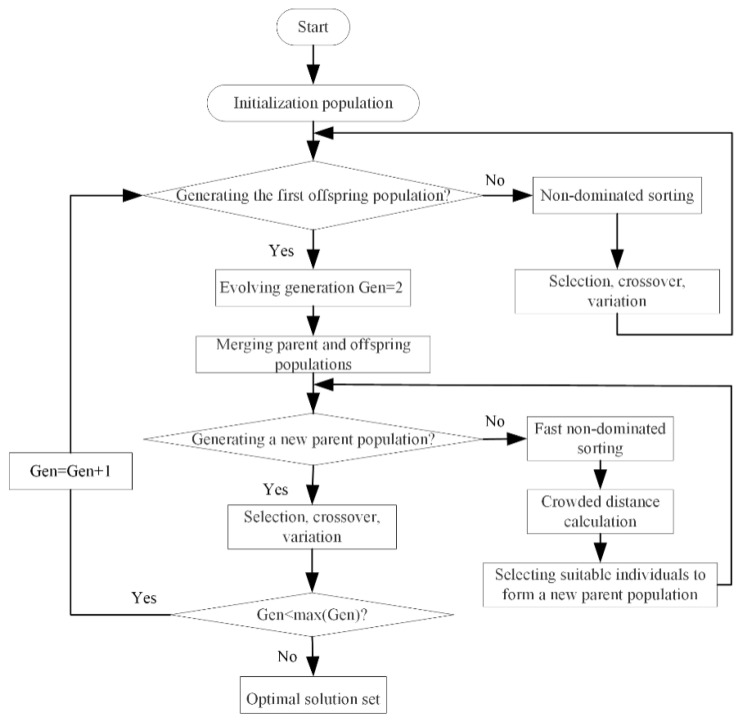
Flow chart of the non-dominated sorting genetic algorithm-II (NSGA-II).

The NSGA-II algorithm [51] is a MOO algorithm based on genetic algorithm, which is based on Pareto optimal solution. When the γ is used as the optimization variable, the dimensionless PO, TE, dimensionless PD, and dimensionless EF cannot be optimized at the same time. Pareto put forward the concept of Pareto domination in 1986. It is impossible to optimize the solution for any objective without making other objectives worse. Since there is no optimal solution to make multiple objectives reach the optimal at the same time, the MOO algorithm gives a series of non-inferior solutions. Compared with other solutions, these non-inferior solutions have the least conflict of objectives, which can provide a better choice space for decision makers. These solution sets are called the optimal Pareto solution sets, and the corresponding objective functions are called the Pareto frontier. The specific algorithm flow chart is shown in Figure 9. There are multiple feasible optimal solutions in Pareto frontier. The decision-making methods such as linear programming technique for multidimensional analysis of preference (LINMAP), technique for order preferences by similarity to ideal solution (TOPSIS) and Shannon entropy are used to select the suitable solution from Pareto frontier. According to Reference [51], the deviation index D is introduced to select the most suitable method.

**Figure 10:**
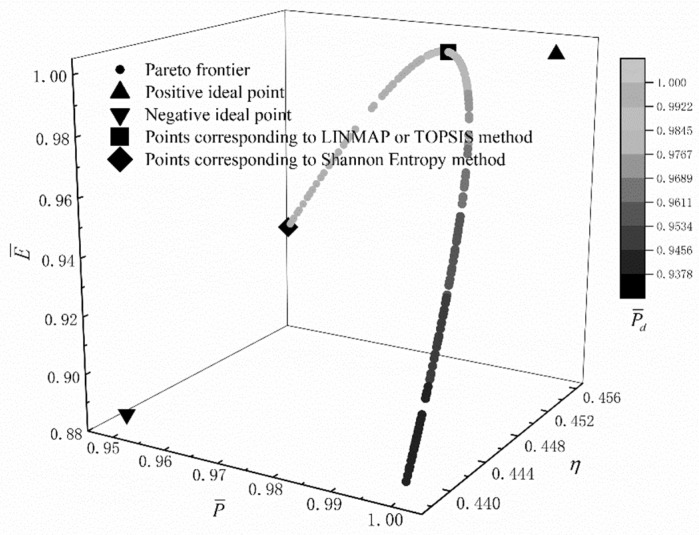
Pareto frontier and optimal solutions for multi-objective optimization.

In order to obtain the optimization design variable of the cycle, the program is conducted by using the “gamultiobj” function of MATLAB. Setting the population “populationsize” as 500 and the algebra “generations” as 1000, the Pareto frontier corresponding to the MOO and the optimal solutions by using three decision-making methods are obtained, as shown in Figure 10. The color on the Pareto frontier edge indicates the size of ${\bar{P}}_{d}$. The positive triangle represents the positive ideal point, the inverted triangle represents the negative ideal point, and the square represents the point corresponding to the LINMAP and TOPSIS decision-making method (the optimal points are the same); the diamond represents the point corresponding to the Shannon entropy decision-making method. According to Figure 10, one can see that, with the increase of TE, the dimensionless PO decreases, the dimensionless PD increases, and the dimensionless EF first increases and then decreases. There is no point on the Pareto frontier which will make the dimensionless PO, TE, dimensionless PD, and dimensionless EF reach the maximum values at the same time, i.e., the Pareto frontier does not include positive ideal point.

Table 1 shows the comparisons of the optimal solutions gained by using the MOO to optimize the performance of the irreversible AC model with $\bar{P}$, η, ${\bar{P}}_{d}$, and $\bar{E}$ as the optimization objectives. It can be seen, from Table 1, that the results gained by using LINMAP and TOPSIS decision-making methods are the same. Compared with results gained by using Shannon entropy decision-making method, the optimal compression ratios gained by using LINMAP and TOPSIS decision-making methods are smaller. The D by using Shannon entropy decision-making method is the largest. In the actual decision-making process, the optimal decision-making method should be selected according to different design requirements.

## 5. Conclusions

Based on the irreversible AC model with constant specific heat established in Reference [28], the effects of cycle temperature ratio, HTL, FL, and IIL on PD were analyzed, and the optimization results under maximum PO and maximum PD were compared. By using the NSGA-II algorithm and taking γ as the optimization variable, the corresponding Pareto frontiers with the dimensionless PO, TE, dimensionless PD, and dimensionless EF as the optimization objectives were obtained. The results show that:

(1) The relationship curve of cycle ${\bar{P}}_{d}-\gamma$ is parabolic-like one. There is an optimal γ which can maximize the PD. With the decrease of τ and the increases of FL and IIL, the PD of cycle decreases.
(2) The relationship curve of cycle ${\bar{P}}_{d}-\eta$ is loop-shaped one. With the decrease of γ and the increases of three losses, the corresponding TE at the maximum PD decreases.
(3) The efficiency $\eta_{{\bar{P}}_{d}}$ under the condition of ${\left(P_{d}\right)}_{\max}$ is larger than the efficiency $\eta_{\bar{P}}$ under the condition of $P_{\max}$, and the corresponding ${\left(v_{4}/v_{1}\right)}_{{\bar{P}}_{d}}$ is smaller than ${\left(v_{4}/v_{1}\right)}_{\bar{P}}$. The AC engine designed under the condition of ${\left(P_{d}\right)}_{\max}$ has smaller size and higher TE.
(4) For the results by using MOO, with the increase of TE, the dimensionless PO decreases, the dimensionless PD increases, and the dimensionless EF first increases and then decreases. There is no point on the Pareto frontier which will maximize $\bar{P}$, η, ${\bar{P}}_{d}$ and $\bar{E}$, i.e., the positive ideal point is not on Pareto frontier.
(5) The suitable solution can be gained by using LINMAP, TOPSIS, and Shannon entropy decision-making methods from Pareto frontier.

## Figures and Tables

**Table 1:** Comparison of multi-objective optimal solutions of the model with $\bar{P}$, η, ${\bar{P}}_{d}$, and $\bar{E}$ as optimization objectives.

Optimization Methods	Decision Methods	Optimization Variables	Optimization Objectives				Deviation Index
		γ	$\bar{P}$	η	${\bar{P}}_{d}$	$\bar{E}$	D
**Four-objective optimization**	LINMAP	6.296	0.984	0.453	0.987	0.999	0.135
	TOPSIS	6.296	0.984	0.453	0.987	0.999	0.135
	Shannon Entropy	7.709	0.949	0.455	1.000	0.924	0.543
Positive ideal point		——	0.999	0.455	1.000	0.999	——
Negative ideal point		——	0.949	0.438	0.938	0.884	——

## Nomenclature

A	Heat released rate by fuel (W)
B	Heat transfer loss coefficient (W/K)
$C_{p}$	Specific heat at constant pressure (J/(mol⋅K))
$C_{v}$	Specific heat at constant volume (J/(mol⋅K))
E	Ecological function (W)
k	Specific heat ratio
$\dot{m}$	Molar flow rate (mol/s)
P	Power output (W)
$P_{d}$	Power density (${W/m}^{3}$)
Q	Quantity of heat transfer rate (W)
T	Temperature (K)
Greek symbol	
γ	Compression ratio (-)
τ	Temperature ratio (-)
η	Thermal efficiency (-)
$\eta_{e}$	Expansion efficiency (-)
$\eta_{c}$	Compression efficiency (-)
μ	Friction coefficient (kg/s)
σ	Entropy generation rate (W/K)
Subscripts	
in	Input
leak	Heat leak
max	Maximum value
out	Output
$\bar{P}$	Max power output condition
${\bar{P}}_{d}$	Max power density condition
pq	Influence of working fluid exhausting to environment
q	Influence of heat transfer loss
μ	Influence of friction Loss
η	Max thermal efficiency condition
0	Environment
1−4,2s,4s	Cycle state points
Superscripts	
—	Dimensionless

## Abbreviations

AC	Atkinson cycle
EF	Ecological function
FL	Friction loss
FTT	Finite time thermodynamics
HTL	Heat transfer loss
IIL	Internal irreversibility loss
MOO	Multi-objective optimization
PD	Power density
PO	Power output
TE	Thermal efficiency
WF	Working fluid
